# Bayesian additional evidence for decision making under small sample uncertainty

**DOI:** 10.1186/s12874-021-01432-5

**Published:** 2021-10-25

**Authors:** Arjun Sondhi, Brian Segal, Jeremy Snider, Olivier Humblet, Margaret McCusker

**Affiliations:** 1 Flatiron Health, Inc., 233 Spring St, New York, NY 10013 USA; 2 Present affiliation: Indigo Ag, Boston, MA 02129 USA; 3 Present affiliation: GRAIL, Inc., Menlo Park, CA 94025 USA

**Keywords:** Real world, Real world evidence, Small sample size, Bayesian

## Abstract

**Background:**

Statistical inference based on small datasets, commonly found in precision oncology, is subject to low power and high uncertainty. In these settings, drawing strong conclusions about future research utility is difficult when using standard inferential measures. It is therefore important to better quantify the uncertainty associated with both significant and non-significant results based on small sample sizes.

**Methods:**

We developed a new method, Bayesian Additional Evidence (BAE), that determines (1) how much additional *supportive* evidence is needed for a non-significant result to reach Bayesian posterior credibility, or (2) how much additional *opposing* evidence is needed to render a significant result non-credible. Although based in Bayesian analysis, a prior distribution is not needed; instead, the tipping point output is compared to reasonable effect ranges to draw conclusions. We demonstrate our approach in a comparative effectiveness analysis comparing two treatments in a real world biomarker-defined cohort, and provide guidelines for how to apply BAE in practice.

**Results:**

Our initial comparative effectiveness analysis results in a hazard ratio of 0.31 with 95% confidence interval (0.09, 1.1). Applying BAE to this result yields a tipping point of 0.54; thus, an observed hazard ratio of 0.54 or smaller in a replication study would result in posterior credibility for the treatment association. Given that effect sizes in this range are not extreme, and that supportive evidence exists from a similar published study, we conclude that this problem is worthy of further research.

**Conclusions:**

Our proposed method provides a useful framework for interpreting analytic results from small datasets. This can assist researchers in deciding how to interpret and continue their investigations based on an initial analysis that has high uncertainty. Although we illustrated its use in estimating parameters based on time-to-event outcomes, BAE easily applies to any normally-distributed estimator, such as those used for analyzing binary or continuous outcomes.

**Supplementary Information:**

The online version contains supplementary material available at 10.1186/s12874-021-01432-5.

## Background

In scientific research, statistical inference is crucial to drawing robust conclusions from data. This is often done through testing a parameter estimate for “statistical significance”, using *p*-values and confidence intervals. These quantities are strongly dependent on sample size; in precision oncology, datasets for rare diseases or biomarker-defined cohorts are often small. This leads to difficulties in deriving insight from analytic results by using standard statistical inference tools.

The primary issue with small sample sizes is that they lead to a lack of statistical power in analyses, meaning that the probability of declaring a true effect or association as statistically significant is small. Due to well-known publication bias, and conflation of “absence of evidence” with “evidence of absence”, non-significant findings are often not reported or published at all [1]. As such, there is no opportunity to learn from the analysis conducted. Even if reported, such findings are usually qualified as “trending towards” or “approaching” significance, which is an arbitrary designation; it does not inform how likely the hypothesis of interest is, or whether future research is worthwhile.

Even a statistically significant result may have high uncertainty, and be unconvincing on its own if derived from a small dataset. This is particularly germane given recent attention on the “replicability crisis” in science [2]. In this scenario, it would be important to ensure the finding is not spurious. Standard analyses do not directly determine how likely the result would be to hold in future studies. Hence, there is a need for statistical tools that make it easier to derive utility from small sample datasets. In this area, Gelman and Carlin (2014) introduced the concept of Type S (sign) and M (magnitude) errors, which quantify the probability of an estimate being in the wrong direction and the expected factor by which its magnitude is exaggerated, conditional on being statistically significant [3]. Segal (2021) also derived confidence intervals for the probability that a replication study would yield estimates more extreme than a certain value (such as the statistical significance threshold) [4]. These methods improve upon standard inference towards the goal of replicable scientific results.

In this article, we introduce a new method, Bayesian Additional Evidence (BAE), for better quantifying the uncertainty of statistical inference output. Our goal is to aid researchers to better interpret results from analyses of small datasets and make decisions about the value of further pursuing research questions. BAE is based on the Bayesian analysis framework, but does not require explicitly setting a prior distribution, and is similar to the recently proposed Analysis of Credibility (AnCred) approach [5]. To implement the BAE approach, we illustrate how to “invert” Bayesian posterior computations to (1) assess the robustness of a significant result, and (2) determine the evidence gap given a non-significant result. Specifically, given an observed parameter estimate and standard error, BAE computes the range of parameter estimates that would need to be observed in a follow-up study in order to make a certain conclusion either in favor or against the hypothesis of interest.

The rest of this paper is structured as follows: in Section 2, we describe the Bayesian normal-normal model, on which our method is based. We then introduce BAE, and illustrate how to use it for decision making given significant or non-significant inferential results. In Section 3, we apply our method to a comparative effectiveness analysis of two treatments for a biomarker-defined cohort from an oncology electronic health record-derived de-identified database, and report the results. We conclude with a brief discussion and conclusion in Section 4 and 5.

## Methods

For the purposes of illustration, we will assume that the goal of the analysis is to estimate *β*_*true*_, the log hazard ratio comparing two treatment arms, adjusting for relevant covariates. We further assume that this parameter is estimated through a standard Cox proportional hazards model, as is common practice in clinical research. However, the methods detailed are applicable to any estimand with a normally-distributed estimator. We also define a statistically significant result as one where the 95% confidence interval excludes the null value, though other significance levels may be used.

The methods we describe are based around Bayesian analysis, and the concept of incorporating prior information to improve precision in estimation. Specifically, we use the concept of “inverting” Bayes’ Theorem, or computing priors that would result in specific posterior distributions of interest.

### Bayesian normal-normal model

A Bayesian analysis computes a *posterior* distribution for *β*_*true*_ (which is treated as a random variable), based on the distribution of the *observed* estimator and a pre-specified *prior* distribution for the true parameter. For our methods, we consider a normally distributed estimator, and a normal prior distribution; this is known as the Bayesian normal-normal model.

We begin with the asymptotic normal distribution of the Cox model estimator $\hat{\beta}$ (Andersen and Gill, 1982) [6], which is:

$$\hat{\upbeta}\left|{\upbeta}_{true}\right.\sim N\left({\upbeta}_{true},\kern0.5em \frac{\sigma }{\sqrt{n}}\right)$$

where *n* is the sample size, and *σ* is the standard deviation of the estimator.

Then, we can assume a normal prior for *β*_*true*_: ~ *N*(µ, *s* )

By conjugacy of the normal-normal model, we then have a closed form for the posterior distribution:

$${\upbeta}_{true}\left|\hat{\upbeta}\right.\kern0.5em \sim \kern0.5em N\left({\upmu}_p,\kern0.5em {S}_p\right)$$

where

$${\displaystyle \begin{array}{c}{S}_p\kern0.5em =\kern0.5em \sqrt{{\left(\frac{n}{\sigma^2}\kern0.5em +\kern0.5em \frac{1}{s^2}\right)}^{-1}}\\ {}{\upmu}_p\kern0.5em =\kern0.5em {S_p}^2\kern0.5em \left(\frac{\upmu}{s^2}\kern0.5em +\kern0.5em \frac{n\hat{\upbeta}}{\sigma^2}\right)\end{array}}$$

Based on the posterior, we can calculate a 95% Bayesian *credible interval* as *μ*_*P*_ ± 1.96 *s*_*P*_. The posterior mean can be interpreted as a weighted average of the prior and observed means, based on how much confidence we have in both quantities. Therefore, if *n* is low (implying more uncertainty in the observed data estimator) and *s* is also low (implying high confidence in the prior), then the posterior mean will be pulled towards *μ*. Conversely, as *n* increases, the posterior mean will tend towards $\hat{\beta}$.

The posterior *precision*, which is defined as the reciprocal of the variance, is equal to the sum of the prior and observed precision. Therefore, the posterior variance will always be less than (or equal) to the observed variance, and so a Bayesian credible interval will necessarily be tighter than the corresponding frequentist confidence interval (or the same width).

Note that this model assumes that the standard deviation *σ* is known, which is often not true in practice. In the Cox model setting, *σ* is a function of *β*_*true*_, which is also unknown. For the implementation of our method described below, we use the estimate $\hat{\sigma}\left(\hat{\beta}\right)$ as a plug-in, which should provide a reasonable estimate. A fully Bayesian analysis would define an additional prior distribution for *σ*.

### Bayesian additional evidence (BAE)

The goal of our proposed method, leveraging the Bayesian framework, is to answer one of two potential questions:

1. Given a non-significant frequentist inferential result, how much additional *supportive* evidence is needed to result in Bayesian posterior credibility?
2. Given a significant frequentist inferential result, how much additional *opposing* evidence is needed to render the result non-credible in the posterior?

For illustration, we start by considering the first question. Suppose we have computed a normally distributed test statistic, with a 95% confidence interval that includes the null value. BAE then searches over prior distributions, which represent potential *future* data, to determine which posterior results provide credible evidence against the null hypothesis. Specifically, we fix the prior standard deviation and search over prior means. The direction of where to search depends on the hypothesis (i.e., is a parameter value greater than or less than the null value of substantive interest?) and is specified by the analyst.

**Fig. 1:**
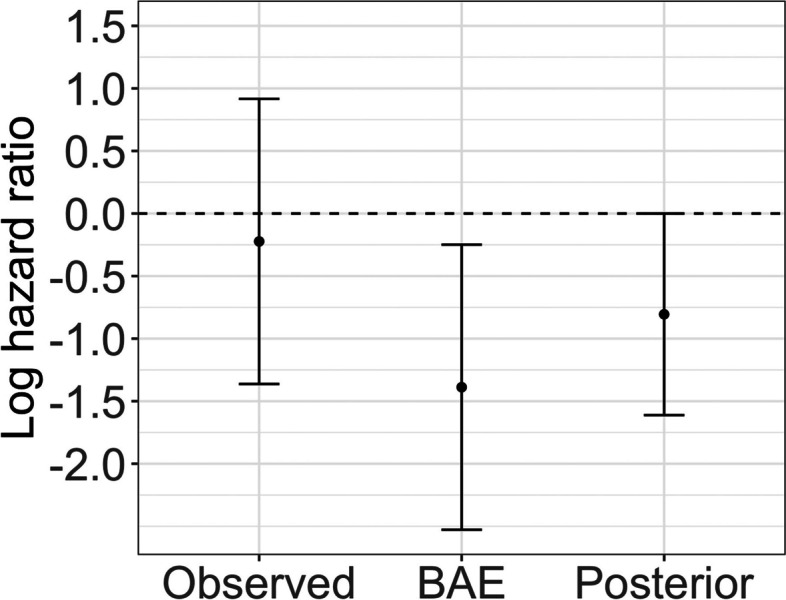
Illustration of the BAE method, given an initial non-significant result, where the hazard ratio confidence interval includes the null value of 1. Assuming the same standard error in a follow-up study, BAE outputs the tipping point hazard ratio estimate that would result in a posterior 95% credible interval that just touches the null value at the upper limit. Therefore, observing the tipping point hazard ratio or anything lower would yield posterior credibility

The output of the BAE method is then the “tipping point” or least extreme prior mean *μ*_∗_ that results in a posterior credible interval that includes the null value. Therefore, **all prior means that are more extreme than** *μ*_∗_ will result in posterior credible intervals that exclude the null value, yielding 95% credibility. This is depicted visually in Fig. 1.

In other words, this method quantifies what type of additional result is needed to have sufficient evidence for an effect. If the returned tipping point is too extreme, then that indicates there is not much evidence for an effect. However, if the tipping point is within a plausible range (based on scientific domain knowledge), then we can declare that the analysis is worthy of follow-up. Although requiring some knowledge of plausible effect sizes, this method does not assume a fully known prior distribution.

In the normal-normal posterior, the prior and the observed quantities are symmetric. Therefore even though we are computing the “prior” mean which leads to a particular posterior, we can think of our initial result as the true prior study in time, and consider the BAE output as the range of effect sizes that would need to be observed in a *future* study in order to achieve a posterior credible interval that excludes the null. We are essentially encoding a replication follow-up study which results in sufficiently credible evidence as our prior.

The interpretation of the BAE output is also dependent on the prior standard deviation used. For example, if we use the same estimated standard error from our frequentist analysis, we can interpret the result as “in a future study with the same level of precision as our current analysis, this is the range of estimates that would yield a posterior credible interval that excludes the null”. We can also decrease or increase the prior standard deviation to encode future studies having higher or lower precision. For example, assuming that *σ* stays constant as *n* changes implies that dividing the observed standard error by $\sqrt{X}$ corresponds to the standard error that would be observed in a study with *X* times more subjects. Due to the dependency of *σ* with *β*_*true*_ in the Cox model setting, this does not hold exactly, but can be used as an approximate heuristic.

With respect to the second question, given a significant initial result, this method can be inverted to see the range of prior means which would make the result non-credible. Here, the output of BAE is then the least extreme prior mean *μ*_∗_ that results in a posterior credible interval that includes the null value. Therefore, **all prior means that are larger in magnitude than** *μ*_∗_ will either result in posterior credible intervals that include the null value yielding non-credibility, or provide credible evidence for an effect in the opposite direction.

Interpretations of BAE applied in either scenario can be found in Table 1 below. We assume that the standard frequentist estimator has been computed, with confidence interval and *p*-value.

**Table 1:** How to apply and interpret BAE given significant or non-significant results from an initial analysis

**Frequentist inference significant**	**Question**: The analysis provides evidence of an effect. How robust would this evidence be given additional data? **BAE output**: BAE provides the range of results that would yield *non-credibility* from a hypothetical follow-up study. If the tipping point is close to the null, then the evidence provided by the current analysis is strong. **Conclusion**: If the range beyond the tipping point is considered plausible, then the current evidence is not strong enough.
**Frequentist inference non-significant**	**Question:** The analysis does not provide strong evidence of an effect. Is it worthwhile to obtain more data for further study? **BAE output:** BAE provides the range of results that would need to be observed in a follow-up study in order to yield *credible evidence* of an effect. If the tipping point is close to the null, then the evidence provided by the current analysis is strong. **Conclusion:** If the range beyond the tipping point is considered plausible, then we can declare the observed data to be useful, and might perform a follow-up study.

Finally, although the BAE tipping point does not have a closed-form solution, implementation is straightforward, and only requires using a root-finding algorithm. Example R code is available in the supplementary material. BAE can also be extended to more complex estimators and prior distributions, so long as the posterior can be computed within the tipping point search algorithm.

### Study design and data sources

A study by Innocenti et al. (2019) aimed to identify genomic factors associated with overall survival (OS) in metastatic colorectal cancer (mCRC) patients treated with either fluorouracil and leucovorin plus oxaliplatin (FOLFOX) or irinotecan (FOLFIRI) chemotherapy and either bevacizumab or cetuximab in the first line (1 L) setting [7]. The authors analyzed data from primary tumor DNA for 843 patients from a larger phase III trial. While the original trial found no statistically significant difference in OS between the treatment arms, the authors reported a very strong clinical benefit of bevacizumab compared to cetuximab (HR = 0.13, 95% CI [0.06, 0.30]) among 37 patients who were known to have microsatellite instability-high (MSI-H) tumors. Although statistically significant, this result is based on a small sample size, with 21 patients receiving bevacizumab and 16 patients receiving cetuximab. The authors also note the potential for selection bias due to MSI-H status not being available for all patients in the original trial. Therefore, it is of interest to attempt a replication of this result with a different dataset in order to confirm this finding.

We compared OS for these two regimens for relevant patients with mCRC from the nationwide Flatiron Health EHR-derived de-identified database. This longitudinal database is comprised of de-identified patient-level structured and unstructured data, curated via technology-enabled abstraction [8, 9]. During the study period, the de-identified data originated from approximately 280 US cancer clinics (~ 800 sites of care). The majority of patients in the database originate from community oncology settings; relative community/academic proportions may vary depending on study cohort. Survival analysis was conducted using a composite mortality variable that aggregates EHR-derived data (structured and unstructured) with links to the SSDI and obituary data [10].

Specifically, we selected a cohort of patients with mCRC diagnosed between 2013 and 2020 who had microsatellite instability high (MSI-H) tumors, and were treated in the 1 L setting with FOLFOX or FOLFIRI chemotherapy plus either bevacizumab or cetuximab (Supplemental Fig. 1). Follow-up began on the start date of 1 L treatment, and ended at the earliest of either date of death or last confirmed structured EHR activity (e.g., non-cancelled medication orders, medication administrations, or clinic visits with vital signs measured). We excluded patients who had a gap of more than 90 days between their mCRC diagnosis date and their first confirmed structured EHR activity date in the Flatiron Health network. We also excluded patients whose date of death was prior to their recorded 1 L start or MSI test result date; such inconsistencies can occur with real world EHR-derived data [10].

The conditional association between OS and 1 L treatment was assessed by fitting a Cox proportional hazards model, adjusting for age, sex, race (dichotomized to White or non-White), BRAF mutation status (present or absent before start of 1 L therapy), and KRAS or NRAS mutation status (present or absent before start of 1 L therapy). Risk set adjustment was applied in order to account for the delayed entry of patients who received their MSI test after the start of 1 L therapy. This analysis is as similar as possible to that conducted by Innocenti et al., (2019) though we were not able to adjust for tumor location, number of metastatic sites, and synchronous or metachronous metastases since those data were not part of the core Flatiron Health data model.

Institutional Review Board approval of the study protocol was obtained prior to study conduct, and included a waiver of informed consent.

## Results

After applying the selection criteria for our study, there were 118 patients in the bevacizumab cohort and 7 patients in the cetuximab cohort. Table 2 provides a description of baseline patient characteristics. Note that due to the small size of the cetuximab arm, we expect there to be high uncertainty in the estimation of the hazard ratio comparing treatments, making this analysis relevant to our method.

**Table 2:** Baseline characteristics of MSI-H mCRC patient cohort

Characteristic		Chemotherapy and bevacizumab *n* = 118	Chemotherapy and cetuximab *n* = 7
Median age at 1 L start, years (IQR)		62 (50, 73)	65 (60, 76)
Sex, n (%)	Female	66 (56)	6 (86)
	Male	52 (44)	1 (14)
Race, n (%)	White	85 (72)	5 (71)
	Other	14 (12)	1 (14)
	Black/Afr. Am.	9 (7.6)	0
	Unknown	7 (5.9)	1 (14)
	Asian	3 (2.5)	0
*BRAF* mutation		26 (22)	3 (43)
*NRAS* or *KRAS* mutation		15 (13)	0
Chemotherapy	FOLFOX	89 (75)	3 (43)
	FOLFIRI	29 (25)	4 (57)

Fitting a Cox model, we estimate that the adjusted hazard ratio of death for patients treated with 1 L chemotherapy plus bevacizumab compared to patients only treated with 1 L chemotherapy plus cetuximab is 0.42 (95% CI: 0.14, 1.23), with a *p*-value of 0.11. Therefore, we cannot conclude that this association is statistically significant at the 5% significance level.

**Fig. 2:**
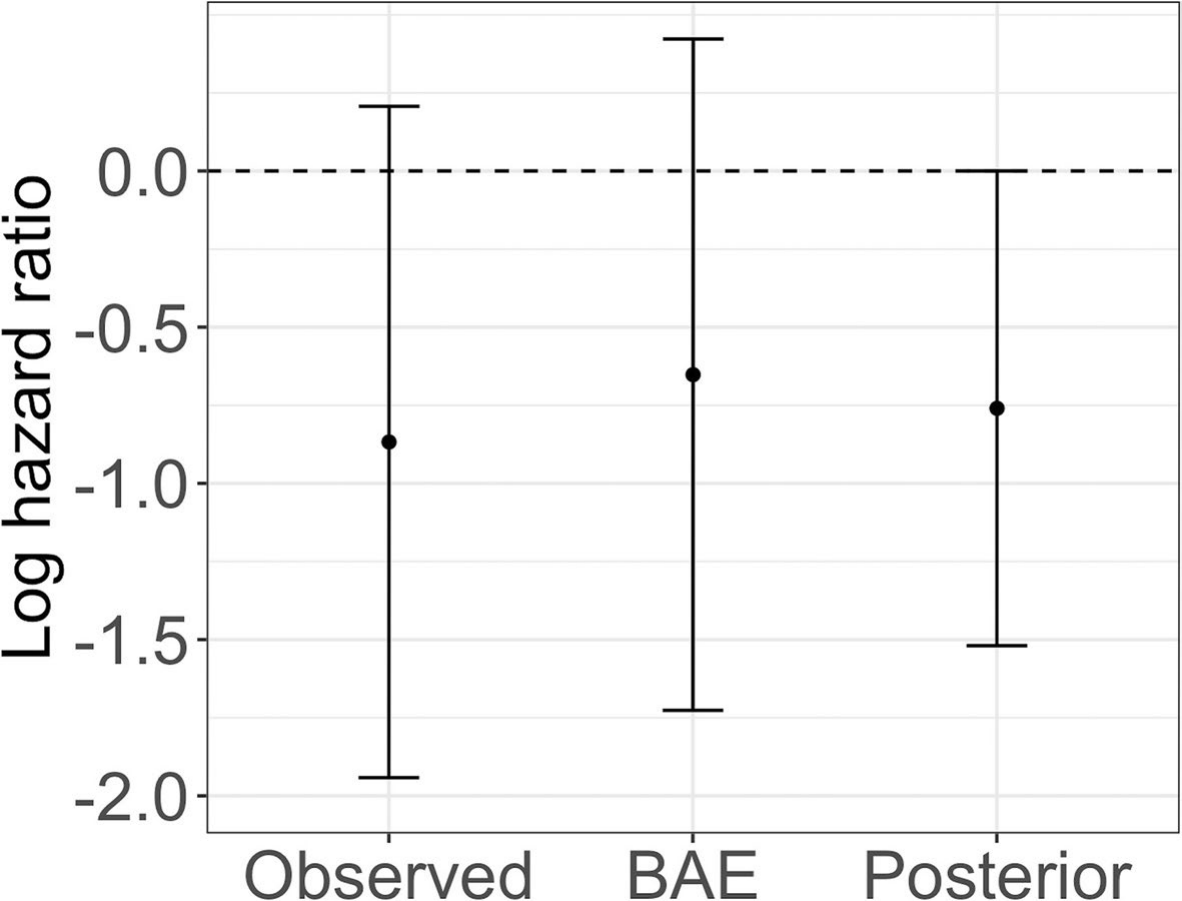
BAE output of log hazard ratio and 95% confidence interval (assuming equal standard error to that observed in study) that would need to be observed in order to yield a posterior distribution with 95% credible interval that touches the null value

Although we observed a non-significant result, due to the small sample size in the chemotherapy plus cetuximab arm, it is important to quantify the uncertainty in the analysis beyond standard methods. We computed the Bayesian Additional Evidence from the frequentist regression analysis. Using the estimated standard error from this analysis as the prior standard deviation, the BAE tipping point is 0.52 on the hazard ratio scale. Therefore, given a replication study with the same level of precision, an observed hazard ratio of 0.52 or smaller would result in posterior credibility for the association of interest. This is depicted graphically in Fig. 2. Scientifically, such hazard ratios would not be considered extreme enough to be implausible. Recall that the similar analysis by Innocenti et al. (2019) reported a hazard ratio of 0.13 with 95% confidence interval (0.06, 0.30) [7]. Therefore, we can conclude that it is worth gathering more evidence for this research question.

The BAE output also tells us that using the published result of Innocenti et al. (2019) as a prior combined with our observed result in a Bayesian analysis would result in 95% posterior credibility. This is because the standard error associated with the prior result is less than that estimated by our analysis. Therefore, given the BAE tipping point of 0.45, a prior mean of 0.13 results in a Bayesian analysis that yields posterior credibility.

## Discussion

It is difficult to extract useful statistical inference from small sample datasets. In this paper, we developed a new approach, for interpreting the evidence provided by these datasets. This is motivated by settings involving rare diseases or biomarkers, where conducting a well-powered study is difficult. Although our method uses the Bayesian framework, it does not require an explicit prior distribution; only domain knowledge of reasonable effect sizes is needed. BAE thus allows for easy integration of prior knowledge with these analyses in order to inform the value of research questions. BAE helps to interpret high-uncertainty results, which can then result in easier decision-making for researchers on how to conduct future studies. We illustrated this in a real world data example involving a small cohort. Here, a standard frequentist analysis yields a non-significant result, but additional uncertainty quantification using BAE shows that credible evidence is plausible with more data. This evidence gap is shown to be tractable given results from a previously published similar analysis.

Within the literature related to statistical analyses using small samples, a change in analytic method is often proposed. Examples include the use of Fisher’s exact test for contingency tables [11], the Firth bias correction for certain generalized linear models [12], and the generalized log rank test statistic for survival analysis [13]. These methods aim to correct inference when asymptotic results may not hold; moreover, BAE can be applied as a complement to analytic results from these methods. Despite this, these methods alone do not assist in making a decision based on an inferential result with high uncertainty, which would likely still occur. As previously discussed, Bayesian analyses present a potential solution. Given sufficiently strong domain knowledge that can be encoded as a prior distribution, we can reduce analytic uncertainty. Bayesian methods can also accommodate a wide variety of data-generating distributions and analyses. However, selecting an appropriate prior is inherently subjective, and may be difficult in many situations due to a lack of published evidence.

Other work also attempts to solve this problem by improving the understanding of analytic results beyond a dichotomous significance threshold. For example, Blume et al, 2019 propose adapting *p*-values to be based on interval (instead of point) null hypotheses, to estimate the fraction of data-supported hypotheses that are “scientifically null”, without requiring a significance threshold [14]. Similarly, Gannon et al, 2019 define a new type of hypothesis test that minimizes a linear combination of the false positive and false negative rates [15]. Within the classic hypothesis testing framework, Segal, 2021 provides confidence intervals for replication probabilities [16]. These approaches and others (see Wasserstein et al, 2019 for an overview) [17] may be useful in certain scenarios depending on the research goals and prior knowledge available.

Toward the goal of improving decision-making, BAE is closely related to the analysis of credibility (AnCred) approach developed by Matthews et al. (2018) [5], which takes the same general approach of performing an inverse Bayesian analysis to find a prior that yields a specific posterior. As with our method, a statistic based on this prior is then compared to plausible effect sizes to arrive at a decision. Although we find this inverse Bayesian approach to be appealing and a useful way to contextualize inferential results, AnCred is more difficult to interpret than BAE, since it provides intervals of prior effect sizes that are consistent with (non-)credible evidence of effects. In the case of non-significant initial results, these intervals can be wide enough to effectively contain any effect size, which is unhelpful for decision making. On the other hand, BAE outputs a clear evidence threshold that would need to be observed in a follow-up study to make certain conclusions.

A limitation of our method is in using the plug-in estimate $\hat{\sigma}\left(\hat{\beta}\right)$ of the coefficient standard deviation in the future study. A fully Bayesian analysis would also model the relationship between *σ* and *β*_*true*_. However, this would involve selection of appropriate priors and additional computational complexity, while our approximation is very straightforward and fast to use in practice. An R implementation of BAE is available in the online supplement.

The use of BAE is also similar to interim analyses of clinical trials, where posterior distributions are computed (based on a pre-specified prior) in order to determine whether the trial should be stopped early due to clear efficacy or futility. It is important to note, however, that BAE should not be seen as a replacement for confirmatory hypothesis testing e.g. in regulatory settings. Rather, it should be used to inform the utility and design of future confirmatory studies. Although we illustrate our approach with a survival analysis of EHR-derived data, it can also be applied to other analyses of datasets having high uncertainty, when using normally-distributed estimators.

## Conclusions

We have developed a novel method to determine the amount of additional evidence needed for a non-significant result to reach Bayesian posterior credibility, or for a significant result to reach non-credibility. We believe BAE can be used to draw initial conclusions from small datasets, which can then be validated with follow-up confirmatory studies. This approach could mitigate under-reporting of analytic results that are not statistically significant, but are clinically useful.

## Supplementary Information

**Additional file 1.**

**Additional file 2.** (R 2 kb)

## Data Availability

The data that support the findings of this study were originated by Flatiron Health, Inc. In order to comply with legal requirements, and to preserve the de-identification status, these de-identified data may be made available upon request, and are subject to a license agreement with Flatiron Health; interested researchers should contact <DataAccess@flatiron.com> to determine licensing terms.

## Abbreviations

1 L: First line
BAE: Bayesian Additional Evidence
EHR: Electronic health record
MSI-H: Microsatellite instability high
OS: Overall survival
mCRC: Metastatic colorectal cancer
