# Supplementary figures and images for: Bayesian additional evidence for decision making under small sample uncertainty
Source: BMC Med Res Methodol. 2021 Oct 25;21:221. doi: 10.1186/s12874-021-01432-5 (PMC8543928; doi:10.1186/s12874-021-01432-5)

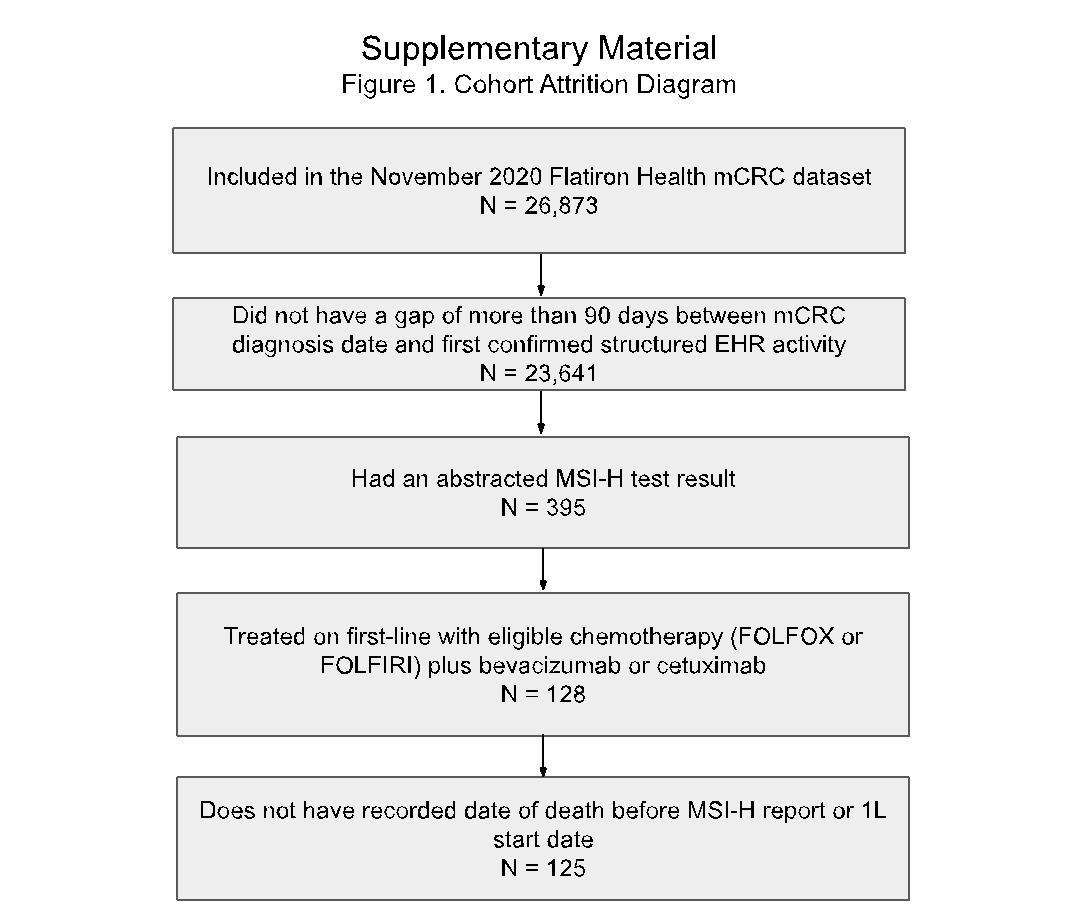

Supplement: Supplementary file 1 — Additional file 1. [file 12874_2021_1432_MOESM1_ESM.docx]
